# Supplementary material for: High lipoprotein(a) concentration is associated with moyamoya disease
Source: Lipids Health Dis. 2024 Jan 22;23:21. doi: 10.1186/s12944-024-02015-1 (PMC10802057; doi:10.1186/s12944-024-02015-1)
Supplement: Supplementary file 1 — Supplementary Material 1: Table S1 Correlation between lipoprotein(a) and the variables. Table S2 Binary logistic regression analysis for MMD and Lp(a) in sensitivity analysis which adjust for statin. Table S3 Binary logistic regression analysis for MMD and Lp(a) in sensitivity analysis which adjust for ASCVD. Table S4 Binary logistic regression analysis for MMD and Lp(a) in sensitivity analysis which adjust for time of inclusion. Table S5 The stratified analysis after adjusting for LDL-C for Lp(a)-cholesterol content, assuming either 17.3%, 30% or 45% mass. Fig. S1 Serum lipoprotein(a) level distribution in moyamoya disease group. Fig. S2 Serum lipoprotein(a) level distribution in control group. Fig. S3 A linear relationship between lipoprotein(a) and moyamoya disease risk by a restricted cubic spine (RCS) [file 12944_2024_2015_MOESM1_ESM.pdf]

Manuscript(11).pdf

WORD COUNT	5476	TIME SUBMITTED	08-JAN-2024 10:58AM
		PAPER ID	105986741

High lipoprotein(a) concentration is associated with moyamoya disease

Abstract

Background: Moyamoya disease (MMD) has attracted the attention of scholars because of its rarity and unknown etiology.

Methods: Data for this study were sourced from the Second Affiliated Hospital of Nanchang University. Regression analyses were conducted to examine the association in Lp(a) and MMD. R and IBM SPSS were conducted.

Results: A cohort comprising 1012 MMD patients and 2024 controls was established through the propensity score matching method. Compared with controls, MMD patients showed higher median Lp(a) concentrations [18.5 (9.6-37.8) mg/dL vs. 14.9 (7.8-30.5) mg/dL, $P < 0.001$]. The odds ratios and 95% confidence intervals for Lp(a) were calculated in three models: unadjusted, model 1 (adjusted for body mass index and systolic blood pressure), and model 2 (adjusted for model 1 plus triglyceride, C-reactive protein, homocysteine, low-density lipoprotein cholesterol). Results were [1.613 (1.299-2.002), $P < 0.001$], [1.598 (1.286-1.986), $P < 0.001$], and [1.661 (1.330-2.074), $P < 0.001$], respectively. Furthermore, age, sex, or hypertension status had nothing to do with this relationship.

Conclusions: Positive relationship exists in Lp(a) and MMD.

Keywords**:** lipoprotein(a); moyamoya disease; risk factor; retrospective cohort study

Introduction

There is a rare but important cerebrovascular disorder known as moyamoya disease (MMD) [1]. It has been reported that MMD is comparatively prevalent in East Asian nations [2, 3]. The two incidence peaks are at approximately 10 years and 30-40 years, with different clinical presentations (intracranial hemorrhage mostly occurs in adult patients, while cerebral ischemia occurs both in pediatric and adult patients) [4]. China is one of the most populous countries, but the diagnosis rate of MMD is lower compared to other East Asian countries [5]. A nationwide retrospective cohort study focused on MMD in China revealed an overall national incidence of 0.59 and a prevalence of 1.01 per 100,000 person-years in 2016 [5]. In recent years, China has witnessed a rise in the diagnostic rate of MMD with continuous enhancements in diagnostic technology [6]. The Lancet Regional Health - Western Pacific recently conducted a study that revealed significant variations in standardized incidence across different regions of China [7]. For instance, Tibet reported a rate of 0.06 [95% confidence intervals (CI), 0.03−0.14], while Jiangxi province had a substantially higher rate of 2.81 (95% CI, 2.65−2.96) for MMD [7]. Emerging studies have noted that the etiology of MMD may involve many molecules associated with inflammation, immunity, and genetic changes [such as Ring Finger Protein 213 (RNF213)] [8]. MMD can be divided into stages I - VI on the basis of Suzuki staging [9]. Different stages may have different inflammation or cytokines involved [10]. Currently, researchers have found that there was a potential relation between MMD and lipoprotein, such as a study found apolipoprotein-E is significantly reduced in MMD cerebrospinal fluid [11]. However, the underlying specific mechanisms of MMD etiology have not been fully

clarified.

A mounting body of evidence substantiates the intricate nexus linking inflammation and Lp(a) [12]. A study has indicated that individuals exhibiting elevated Lp(a) levels experience enhanced arterial inflammation [13]. Clinically, predictors of MMD risk urgently need to be explored. Considering that both Lp(a) and MMD are associated with inflammation, an intrinsic relationship might exist between Lp(a) and MMD. However, no relevant studies have been conducted so far. Therefore, this research was designed to investigate within a comprehensive large case-control study whether Lp(a) and MMD have any correlation.

Methods

Study design and participants

Data from 2007-2021,were retrieved and electronic medical records of individuals hospitalized with a primary diagnosis of MMD in The Second Affiliated Hospital of Nanchang University were reviewed. Patients were not eligible if (1) the value of Lp(a) was not measured or (2) age was < 18 years old. For the control group, data on MMD-free inpatients from 2017 to 2021 with Lp(a) levels were collected. The following patients were excluded from controls: (1) undiagnosed patients, (2) cancer patients, (3) patients with liver and kidney insufficiency, (4) patients of age < 18 years, and (5) patients with infection, poisoning, and other diseases that could affect results. Finally, using propensity score matching (PSM) method, 1,012 patients with MMD and 2,024 controls were matched at a 1:2 ratio for sex, age, smoking status, alcohol consumption status, coronary heart disease (CHD), and hypertension to eliminate confounding factors included in the study (**Fig. 1**).

PSM involves using a statistical model to calculate the comprehensive propensity score of each observation for each covariate and then matching according to whether the propensity score is close [14]. Ethics review and approval were obtained from the ethics committees of the Second Affiliated Hospital of Nanchang University.

Clinical and laboratory variables

Study included the following appropriate basic clinical data: sex, age, weight, body mass index (BMI), alcohol consumption status, systolic blood pressure (SBP), smoking status, and diastolic blood pressure (DBP). Diseases included diabetes mellitus, CHD, and hypertension. Laboratory values included Lp(a), high-density lipoprotein cholesterol (HDL-C), estimated glomerular filtration rate (eGFR), uric acid, albumin, platelet count, glucose, fibrinogen, triglyceride (TG), lymphocyte count, c-reactive protein (CRP), apolipoprotein A-I, apolipoprotein B, total cholesterol (TC), creatinine, neutrophil count, low-density lipoprotein cholesterol (LDL-C), neutrophil-lymphocyte ratio (NLR) and homocysteine (HCY).

Lp(a) measurement

For patients in the MMD group, Lp(a) was measured concurrently during hospitalization when MMD was diagnosed. Two Lp(a) Assay Kits (Shanghai Kehua Biology Inc., China, production batch: 20180212 and Beijing Antu Inc., China, LOT:10723C11) were used as described in a previous study [15]. Both kits used the latex immunoturbidimetric method to detect Lp(a). The preferred sites of monoclonal antibodies are KIV-8 and KIV-9. The calibrators are traceable to WHO/IFCC SRM 2B.

Diagnostic criteria for MMD

i. Digital subtraction angiography (DSA), CT angiography (CTA), or magnetic resonance angiography (MRA) demonstrating stenosis or occlusion in distal segments of the internal carotid artery, anterior cerebral artery, and/or middle cerebral artery; ii. DSA, CTA, or MRA depicting an anomalous vascular network at the skull base; and iii. The above manifestations are bilateral, but the degree of the disease may be different [9, 16].

Statistical analysis

PSM was used to screen the MMD and control groups to compare the clinical variables, achieve the goal of covariate equalization, and reduce bias as in the previous methods [15]. The quantitative variables in this study all showed skewed distributions. Therefore, Mann–Whitney U test was utilized to examine quantitative factors. Chi-square test was implemented to explore qualitative factors. Using the Pearson test, monotonic correlation of Lp(a) with other factors was investigated. The stratification of Lp(a) values involved dividing them into four quartiles for subsequent regression analysis and stratified analysis. Binary logistic regression was utilized. In model 1, adjustments were made for BMI and SBP. In model 2, adjustments included TG, CRP, HCY, and LDL-C, in addition to the factors considered in model 1. In model 3, adjustments were made by including statin along with the factors considered in model 2. Restricted cubic spine (RCS) was conducted to examine the relation in Lp(a) and MMD risk. To eliminate potential confounders [arteriosclerotic cardiovascular disease (ASCVD) and consistent time], sensitivity analyses were performed. Stratified analyses were carried out by age, sex, LDL-C, and hypertension. The adjustment in the analysis involved BMI, SBP, CRP, TG, HCY, and LDL-C in age, sex,

and hypertension groups. Adjustments of LDL-C group were BMI, SBP, CRP, TG, and HCY. The significance level for all tests was 0.05 on a two-sided basis. Analyses in this study mainly relied on R (version 4.1.1) and SPSS (SPSS, Inc., Chicago, Illinois).

Results

Clinical features

Study included 1,012 MMD patients and 2,024 control subjects matched for sex, age, smoking status, drinking status, hypertension, and CHD. A skewed distribution of Lp(a) existed in both the MMD group and controls (**Fig. S1 and S2**). The median Lp(a) values in the MMD and control groups differed significantly [18.5 (9.6-37.8) mg/dL vs. 14.9 (7.8-30.5) mg/dL, $P < 0.001$]· (**Table 1**). MMD patients had a higher SBP, neutrophil count, and eGFR than the control group. Furthermore, compared with the controls, the albumin, platelet count, lymphocyte count, HDL-C, LDL-C, uric acid, apolipoprotein A-I, apolipoprotein B, TC, and creatinine were lower in the MMD patients (**Table 1**), while MMD patients used significantly more statins (50.9% vs. 25.9%, $P < 0.001$).

Lp(a) concentration and other relevant clinical data

(i) significant and positive relation to LDL-C (r: 0.142, $P < 0.001$), TC (r: 0.079, $P < 0.001$), fibrinogen (r: 0.154, $P < 0.001$), platelet count (r: 0.048, $P = 0.048$), and apolipoprotein B (r: 0.116, $P < 0.001$) (**Table S1**); (ii) significant and negative relationships with weight (r: - 0.092, $P < 0.001$), uric acid (r: - 0.065, $P < 0.001$), TG (r: - 0.060, $P = 0.001$), glucose (r: - 0.038, $P = 0.038$), and lymphocyte count (r: - 0.066, $P < 0.001$) (**Table S1**); and (iii) significant and positive relationships with inflammatory markers (including neutrophil

count (r: 0.040, *P* =0.028) and neutrophil-lymphocyte ratio (NLR) (r: 0.073, *P* < 0.001) (**Table S1**).

Association between Lp(a) and MMD

Results revealed that the odds ratios (ORs) and 95% CIs of Lp(a) in unadjusted model, model 1 (BMI and SBP were adjusted), model 2 (model 1 plus LDL-C, TG, HCY, and CRP were adjusted) and model 3 (model 2 plus statin were adjusted) were [1.005 (1.003-1.008), *P* = 0.001], [1.005 (1.003-1.008), *P* < 0.001], [1.006 (1.003-1.009), *P* < 0.001] and [1.005 (1.002-1.007), *P* = 0.001]. Then, Lp(a) was calculated into quartile 1 (Q1, n = 759), 0-8.24 mg/dL; quartile 2 (Q2, n = 759), 8.24-15.94 mg/dL; quartile 3 (Q3, n = 760), 15.94-32.56 mg/dL; and quartile 4 (Q4, n = 758), > 32.56 mg/dL. **Table 2** showed that each Lp(a) quartile 3-4 showed a significantly higher association with MMD than quartile 1 [Q3, 1.458 (1.173-1.813), *P* = 0.001] and [Q4, 1.613 (1.299-2.002), *P* < 0.001] in the unadjusted model. The OR and 95% CIs of Q3-Q4 were [1.443 (1.159-1.795), *P* = 0.001] and [1.598 (1.286-1.986), *P* < 0.001] (BMI and SBP were adjusted in model 1). Model 2 included model 1 plus LDL-C, TG, HCY, and CRP and was adjusted, and the data revealed a substantial relation between a higher Lp(a) value and an advanced risk of MMD [Q4, 1.661 (1.330-2.074), *P* < 0.001]. Furthermore, to explore whether statin use affects outcomes, model 3 plus statin was adjusted, and the results suggested that statin use did not influence the association between Lp(a) and MMD [Q4, 1.524 (1.212-1.916), *P* < 0.001] (**Table S2**). To further clarify the authenticity of the results of binary conditional logistic regression models, RCS was carried out (**Fig. S3**). P-Nonlinear was 0.096. When the hazard ratio/OR=1, the value of Lp(a) was 15.865 (the value was within Q2). It was found that Lp(a) and MMD

risk were linearly related.

Sensitivity analysis was used to eliminate potential confounders. First, considering that ASCVD would affect Lp(a) or the MMD diagnosis, a sensitivity analysis that excluded ASCVD patients from the MMD group and control group was used. The OR and 95% CIs of Lp(a) in unadjusted model, model 1 and model 2 were [1.006 (1.003-1.008), $P = 0.001$], [1.006 (1.003-1.008), $P < 0.001$], and [1.006 (1.004-1.009), $P = 0.001$]. On the basis of Lp(a) levels, four quartiles were calculated: quartile 1 (Q1, n = 708), 0-8.17 mg/dL; quartile 2 (Q2, n = 708), 8.17-15.90 mg/dL; quartile 3 (Q3, n = 708), 15.90-32.10 mg/dL; and quartile 4 (Q4, n = 708), > 32.10 mg/dL. The OR and 95% CIs of Lp(a) in unadjusted model, model 1 and model 2 were [1.602 (1.281-2.005), $P < 0.001$], [1.596 (1.274-1.998), $P < 0.001$] and [1.671 (1.327-2.104), $P < 0.001$] (**Table S3**). Second, considering the different years of inclusion of patients between the two groups, a sensitive analysis that removed the 332 patients from 2006-2016 in the MMD group to keep the time consistent was conducted. Then, 1:2 matching according to the original PSM method was conducted. The OR and 95% CIs of Lp(a) in unadjusted model, model 1 and model 2 were [1.004 (1.001-1.008), $P = 0.004$], [1.004 (1.001-1.007), $P = 0.005$], and [1.005 (1.002-1.009), $P = 0.001$]. Four quartiles were created based on Lp(a) levels: quartile 1 (Q1, n = 510), 0-7.80 mg/dL; quartile 2 (Q2, n = 510), 7.80-15.09 mg/dL; quartile 3 (Q3, n = 511), 15.09-32.09 mg/dL; and quartile 4 (Q4, n = 509), > 32.09 mg/dL. Then, binary conditional logistic regression of Lp(a) quantiles and MMD risk was analyzed (**Table S4**), and it showed the same results as previously. The OR and 95% CIs of Lp(a) in unadjusted model, model 1 and model 2 were [1.437 (1.107-1.865), $P = 0.006$], [1.434 (1.105-1.862), $P = 0.007$] and [1.513 (1.156-1.980), $P = 0.003$].

Furthermore, stratified analyses were carried out (**Fig. 2**). The Lp(a) quartiles were stratified by age (age > 60: n = 675, age ≤ 60: n = 2361) (**Fig. 2A**), sex (male: n = 1561, female: n = 1475) (**Fig. 2B**), LDL-C (LDL-C ≤ 130 mg/dL: n = 2388, LDL-C > 130 mg/dL: n = 648) (**Fig. 2C**) and hypertension (hypertension: n = 1209, non-hypertension: n = 1827) (**Fig. 2D**) to assess the risk of MMD in each. Separate adjustments were made for age, sex, hypertension (including BMI, SBP, CRP, TG, HCY, and LDL-C), and LDL-C (including BMI, SBP, CRP, TG, and HCY) groups. In **Fig. 2A**, patients aged ≤ 60 showed a critically elevated risk of MMD in Q4 group [1.640 (1.276, 2.107), *P* < 0.001], and the result was consistent in those aged > 60 [Q4, 1.794 (1.101, 2.923), *P* = 0.019]. Significant increase existed in MMD risk in Q4 group in both the male subgroup [1.525 (1.120, 2.076), *P* = 0.007] and the female subgroup [1.781 (1.287, 2.464), *P* < 0.001] (**Fig. 2B**). Furthermore, Q4 of Lp(a) and MMD risk were positively correlated in patients whose LDL-C ≤ 130 mg/dL [1.711 (1.342, 2.182), *P* < 0.001] (**Fig. 2C**). Additionally, the MMD risk was dramatically high in the presence [2.305 (1.614, 3.294), *P* < 0.001] or absence [1.343 (1.004,1.795), *P* < 0.001] of hypertension in the Q4 group (**Fig. 2D**). Considering that Lp(a) has a certain influence on the value of LDL-C, sensitivity analysis of Lp(a)-adjusted LDL-C was used to eliminate potential confounders according to this formula [17]: Lp(a)-adjusted LDL-C = laboratory LDL-C – [Lp(a) mass × 17.3% or 30% or 45%)]. Compared with controls, the median Lp(a)-adjusted LDL-C [30% Lp(a) mass] value was lower in MMD patients (98.10 vs. 101.20. mg/dL, *P* < 0.001). The new stratified analysis revealed that a positive association existed with Q4 and MMD in Lp(a)-adjusted LDL-C ≤ 130 mg/dL group [1.585 (1.253, 2.004), *P* < 0.001], while there was no significance in the

LDL-C >130 mg/dL group [1.045 (0.551, 1.979), $P = 0.893$]. Moreover, when adjusted LDL-C by 17.3% or 45% Lp(a) mass, the results remained consistent (**Table S5**).

Discussion

This is a large case-control study investigating the relation in Lp(a) and MMD. The results demonstrated that Lp(a) had a positive relationship with MMD, Moreover, age, sex, or hypertension status had nothing to do with this relationship.

It is well known that Lp(a) has two components: LDL-like particles (composed of apolipoprotein B, free cholesterol, and phospholipids) and Apo(a) [18]. The proatherogenic and proinflammatory effects of lipid components can obviously promote the accumulation of Lp(a) cholesterol within the vascular wall [19]. These properties of Lp(a) are the molecular basis for the induction of ASCVD, such as CHD and atherosclerosis [20]. The mechanisms of other Lp(a) related diseases consist of three aspects [20]: atherosclerosis; thrombosis; inflammation. At present, there are three possible causes of MMD: endothelial colony-forming cells and growth factors such as VEGF; genetic factors; mechanisms associated with inflammation and immune [8]. Therefore, inflammation might be the mechanism that works for both. For example, one study found that interleukin-1 beta might act as a clinically useful biomarker in MMD [21] and another study found that lipoprotein metabolism might play a critical role in MMD [22]. In addition, by serum proteomic analysis of adult patients with MMD, Wang, Z., et al found that lipoprotein dysfunction might get involved in MMD [23]. These studies revealed the value of in-depth exploration of the connection between lipoprotein and MMD. In an earlier investigation by Angeliki Skardoutsou et al., the Lp(a) value in a child with MMD was

increased. However, due to the limitation that the study analyzed only one specific patient, the data were not sufficient to obtain an actual correlation between Lp(a) and MMD [24].

Certain research has discovered that Lp(a) levels vary by sex and age. Elevated Lp(a) level is related with low type 2 diabetes risk among male individuals and patients over 60 years old [15]. Lp(a) and aortic stenosis (AS) surgery appear to have a correlation in patients over 60, which reveals that age could influence the effect of Lp(a) on AS risk [25]. One study found that only in the female group were the likelihood of atrial fibrillation and Lp(a) values negatively related [26]. Therefore, this study conducted a stratified analysis of four subgroups (age, sex, LDL-C, and hypertension) (**Fig. 2**). High Lp(a) concentration was related to MMD regardless of age or sex. Of note, in the Q4 group, when LDL-C > 130 mg/dL, Lp(a) had no significant relationship with MMD (**Fig. 2C**). The following are potential reasons: First, the sample size of the Q4 group with LDL-C > 130 mg/dL is 199 (MMD group n = 52; control group n = 147); a sample of such small size cannot provide sufficient testing efficiency. Second, people with high LDL-C have more ASCVD [27]. Based on a retrospective analysis of 1522 cases and 1691 controls in a Han population, LDL-C and Lp(a) are additive in initial acute myocardial infarction [28]. Acute myocardial infarction risk resulting from exposure to increased Lp(a) and elevated LDL-C is significantly higher compared to the two cumulative risks associated with each factor individually. Therefore, when LDL-C > 130 mg/dL, superimposed effect of the two might make other vascular diseases more prominent, which is likely to mask the development of MMD (an insidious vascular disease), thereby “impairing” the effect of Lp(a) on MMD. At the same time, small samples definitely make the results less convincing. Therefore,

well-designed studies will be needed to explore this in the future.

Additionally, there are several other risk factors for MMD. HCY is one of them. A study on whether children's thyroid function is associated with MMD that involved 114 children with 114 healthy control subjects revealed that elevated HCY was related to MMD [29]. However, it did not appear that HCY and MMD were associated in any noteworthy way (P = 0.984) in this study. The difference in results is probably attributed to the extremely limited number of participants in their studies (less than 200 patients) relative to the number of participants in this study (more than 1,000 patients). In addition, the control group in this study consisted of inpatients without MMD, while previous studies used healthy people as the control group, which might also contribute to the different results.

Based on prior research findings, the direct cause of MMD is abnormally enhanced arteriogenesis and angiogenesis, thus causing arterial blockage [30]. The development of MMD mainly includes two major parts: arterial injury or occlusion and abnormal angiogenesis. The function of Lp(a) in ASCVD is not only through inflammation but also includes various mechanisms, such as lipid metabolism disorder [19, 31]. Apo(a) is one of the components of Lp(a) and has procoagulant effects, such as inhibiting the activation of fibrinogen, thereby increasing the formation of thrombin [19]. LDL-like particles, another component of Lp(a), may induce the development of foam cells and cause vascular injury [32]. Pirro M. et al. illustrated that Lp(a) may cause the release of inflammatory cytokines, triggering endothelial activation [33], which implied that Lp(a) gets involved in endothelial homeostasis and vascular dysfunction through inflammation. The research of Günter Christ et al. showed that Lp(a) exhibits a specific inhibitory impact on the proliferation of blood

vessels, which is contradictory to the significant blood vessel growth in MMD patients [34]. Although these speculations still need to be confirmed by further research, the link between Lp(a) and MMD is not just an inflammation-related mechanism, given that this study showed a lack of correlation between CRP and Lp(a) (r: 0.007, $P = 0.712$) or MMD (r: -0.025, $P = 0.162$).

Study strengths and limitations

Several strengths can be found in this study. First, compared with similar studies whose sample sizes are almost always less than 500, the most prominent advantage is its large sample size (1012 MMD patients), which makes the conclusion more convincing to some extent. Second, the study found that elevated Lp(a) value is positively related to MMD among Chinese individuals, which had not been mentioned previously. Third, the PSM method and stratified analysis were conducted to eliminate some confounding factors, which minimized the bias caused by confounding factors. However, several limitations also exist. First, since this study was retrospective, it could not demonstrate that Lp(a) is causally correlated with MMD. Future prospective research will need to further confirm whether Lp(a) is related to the clinical presentation of patients with MMD. Second, several residual confounding factors may have been introduced due to the use of inpatient data in this study. Third, the influence of genes was not considered, which might cast doubt on the results. Fourth, it would be of great importance to determine the Lp(a) value in MMD patients depending on their presentation. However, we did not analyze this in the study, which might influence the conclusion. Fifth, the cohort under study is a selected subgroup of MMD patients in whom Lp(a) was measured, thus excluding more than 50%. An

unintended bias may exist related to selecting those MMD whose Lp(a) measurements were thought necessary. Therefore, considering the influences of sex, age, smoking status, alcohol consumption status, CHD, hypertension, and other factors on MMD, the PSM method and stratified analysis were used to eliminate confounding factors, which minimized the bias caused by confounding factors. In addition, the current researchers plan to perform a prospective study and will consider solving the above problems.

Conclusion

With this large case-control study, elevated Lp(a) value is significantly positively associated with MMD, which is not influenced by age, sex, or hypertension. It hints that patients with elevated Lp(a) values might be considered as a potential risk factor for MMD, which deserves future prospective cohort studies.

References

1. Huang, S., et al., Etiology and pathogenesis of Moyamoya Disease: An update on disease prevalence. Int J Stroke. 2017; 12(3): 246-253.
2. Kim, T., et al., Epidemiology of Moyamoya Disease in Korea: Based on National Health Insurance Service Data. J Korean Neurosurg Soc. 2015; 57(6): 390-5.
3. Hayashi, K., et al., An epidemiological survey of moyamoya disease, unilateral moyamoya disease and quasi-moyamoya disease in Japan. Clin Neurol Neurosurg. 2013; 115(7): 930-3.
4. Kim, J.S., Moyamoya Disease: Epidemiology, Clinical Features, and Diagnosis. J Stroke. 2016; 18(1): 2-11.
5. Sun, Y., et al., Incidence and prevalence of moyamoya disease in urban China: a nationwide retrospective cohort study. Stroke Vasc Neurol. 2021; 6(4): 615-623.
6. Bao, X.Y., et al., Epidemiology of Moyamoya Disease in China: Single-Center, Population-Based Study. World Neurosurg. 2019; 122: e917-e923.
7. Zhang, D., et al., Epidemiology of Moyamoya disease in China: A nationwide hospital-based study. Lancet Reg Health West Pac. 2022; 18: 100331.
8. Mineharu, Y. and S. Miyamoto, RNF213 and GUCY1A3 in Moyamoya Disease: Key Regulators of Metabolism, Inflammation, and Vascular Stability. Front Neurol. 2021; 12: 687088.
9. Guidelines for diagnosis and treatment of moyamoya disease (spontaneous occlusion of the circle of Willis). Neurol Med Chir (Tokyo). 2012; 52(5): 245-66.
10. Weinberg, D.G., et al., Moyamoya disease: a review of histopathology, biochemistry, and genetics. Neurosurg Focus. 2011; 30(6): E20.
11. Kashiwazaki, D., H. Uchino, and S. Kuroda, Downregulation of Apolipoprotein-E and Apolipoprotein-J in Moyamoya Disease-A Proteome Analysis of Cerebrospinal Fluid. J Stroke Cerebrovasc Dis. 2017; 26(12): 2981-2987.
12. Hoogeveen, R.C. and C.M. Ballantyne, Residual Cardiovascular Risk at Low LDL: Remnants, Lipoprotein(a), and Inflammation. Clin Chem. 2021; 67(1): 143-153.
13. Lüscher, T.F., Frontiers in lipid research: lipoprotein(a), apolipoprotein C-III and E, and PCSK9 and inflammation. Eur Heart J. 2019; 40(33): 2741-2744.
14. Morgan, C.J., Reducing bias using propensity score matching. J Nucl Cardiol. 2018; 25(2): 404-406.
15. Fu, Q., et al., High lipoprotein(a) concentrations are associated with lower type 2 diabetes risk in the Chinese Han population: a large retrospective cohort study. Lipids Health Dis. 2021; 20(1): 76.
16. Han, D.H., D.H. Nam, and C.W. Oh, Moyamoya disease in adults: characteristics of clinical presentation and outcome after encephalo-duro-arterio-synangiosis. Clin Neurol Neurosurg. 1997; 99 Suppl 2: S151-5.
17. Thayabaran, D., et al., The effect of adjusting LDL-cholesterol for Lp(a)-cholesterol on the diagnosis of familial hypercholesterolaemia. J Clin Lipidol. 2023; 17(2): 244-254.
18. Jawi, M.M., J. Frohlich, and S.Y. Chan, Lipoprotein(a) the Insurgent: A New Insight into the Structure, Function, Metabolism, Pathogenicity, and Medications Affecting Lipoprotein(a) Molecule. J Lipids. 2020; 2020: 3491764.

19. Kamstrup, P.R., Lipoprotein(a) and Cardiovascular Disease. Clin Chem. 2021; 67(1): 154-166.
20. Tsimikas, S., A Test in Context: Lipoprotein(a): Diagnosis, Prognosis, Controversies, and Emerging Therapies. J Am Coll Cardiol. 2017; 69(6): 692-711.
21. Han, W., et al., Association of Brain-Gut Peptides with Inflammatory Cytokines in Moyamoya Disease. Mediators Inflamm. 2020; 2020: 5847478.
22. Ge, P., et al., Modifiable Risk Factors Associated With Moyamoya Disease: A Case-Control Study. Stroke. 2020; 51(8): 2472-2479.
23. Wang, Z., et al., Data-Independent Acquisition-Based Serum Proteomic Profiling of Adult Moyamoya Disease Patients Reveals the Potential Pathogenesis of Vascular Changes. J Mol Neurosci. 2022; 72(12): 2473-2485.
24. Skardoutsou, A., et al., Moya moya syndrome in a child with pyruvate kinase deficiency and combined prothrombotic factors. J Child Neurol. 2007; 22(4): 474-8.
25. Ljungberg, J., et al., Lipoprotein(a) and the Apolipoprotein B/A1 Ratio Independently Associate With Surgery for Aortic Stenosis Only in Patients With Concomitant Coronary Artery Disease. J Am Heart Assoc. 2017; 6(12).
26. Tao, J., et al., Low lipoprotein(a) concentration is associated with atrial fibrillation: a large retrospective cohort study. Lipids Health Dis. 2022; 21(1): 119.
27. Zhang, Y., et al., Association Between Cumulative Low-Density Lipoprotein Cholesterol Exposure During Young Adulthood and Middle Age and Risk of Cardiovascular Events. JAMA Cardiol. 2021; 6(12): 1406-1413.
28. Hu, Y., et al., Interaction of lipoprotein(a) with low-density lipoprotein cholesterol on first incident acute myocardial infarction. Clin Chim Acta. 2020; 501: 1-5.
29. Li, H., et al., Increased thyroid function and elevated thyroid autoantibodies in pediatric patients with moyamoya disease: a case-control study. Stroke. 2011; 42(4): 1138-9.
30. Rafat, N., et al., Increased levels of circulating endothelial progenitor cells in patients with Moyamoya disease. Stroke. 2009; 40(2): 432-8.
31. Boffa, M.B., S.M. Marcovina, and M.L. Koschinsky, Lipoprotein(a) as a risk factor for atherosclerosis and thrombosis: mechanistic insights from animal models. Clin Biochem. 2004; 37(5): 333-43.
32. Sorci-Thomas, M.G. and M.J. Thomas, Microdomains, Inflammation, and Atherosclerosis. Circ Res. 2016; 118(4): 679-91.
33. Pirro, M., et al., Lipoprotein(a) and inflammation: A dangerous duet leading to endothelial loss of integrity. Pharmacol Res. 2017; 119: 178-187.
34. Christ, G., et al., Plasmin activation system in restenosis: role in pathogenesis and clinical prediction? J Thromb Thrombolysis. 1999; 7(3): 277-85.

Figures and Tables

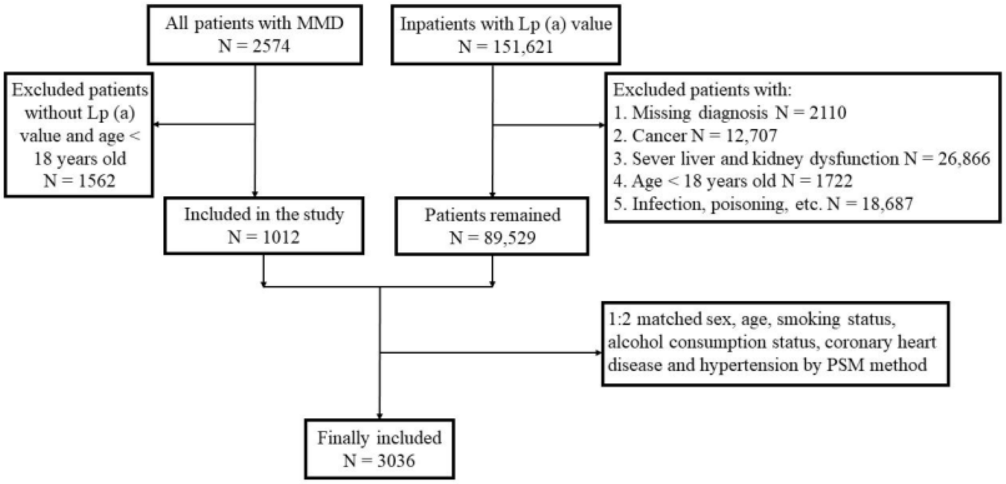

29
Fig. 1. Study flowchart

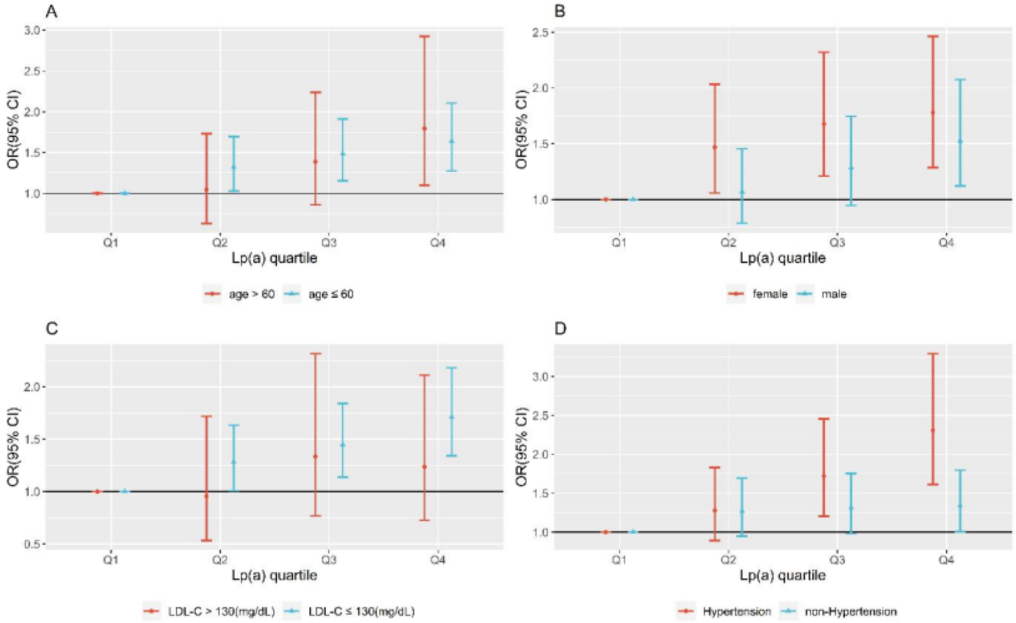

Fig. 2 Stratified analyses for MMD of Lp(a) quartiles by age (A) (age > 60: n = 675, age ≤ 60: 2361), sex (B) (male: n = 1561, female: n = 1475), LDL-C (C) (LDL-C ≤ 130 mg/dL: n = 2388, LDL-C >130 mg/dL: n = 648), and hypertension (D) (hypertension: n = 1209, non-hypertension: n = 1827)

The adjustments of (A), (B), and (D) were BMI, SBP, CRP, TG, HCY, and LDL-C. Adjustment of (C) was BMI, SBP, CRP, TG, and HCY

*The stratified test used binary logistic regression with $P < 0.05$ being meaningful

Table 1. Baseline features after matching

Characteristics	MMD group	Control group	P value
Total number (n)	1012	2024	-
Demographic data			
Male, n (%)	520 (51.5)	1041 (51.4)	0.980‡
Age	51.0 (45.0-60.0)	51.0 (45.0-60.0)	0.981†
BMI (kg/m2)	23.4 (22.4-24.5)	23.5 (21.9-25.3)	0.078†
Smoking status, n (%)	137 (13.5)	271 (13.4)	0.910‡
Alcohol consumption status, n (%)	124 (12.6)	244 (12.1)	0.875‡
SBP (mmHg)	131 (120-145)	128 (117-144)	0.001*†
DBP (mmHg)	78 (70-84)	78 (71-86)	0.017*†
Medical history			
Coronary heart disease, n (%)	35 (3.5)	70 (3.5)	1.000‡
Hypertension, n (%)	402 (39.7)	807 (39.9)	0.937‡
Diabetes mellitus, n (%)	145 (14.3)	349 (17.2)	0.040*‡
Laboratory values			
Lp(a) (mg/dL)	18.5 (9.6-37.8)	14.9 (7.8-30.5)	<0.001*†
Albumin (g/L)	40.9 (37.7-43.0)	41.6 (38.5-44.1)	<0.001*†
Platelet count (10^9/L)	212.0 (177.0-247.0)	216.0 (179.0-257.0)	0.038*†
Glucose (mmol/L)	5.70 (4.99-6.65)	5.54 (4.87-6.60)	0.053†
Fibrinogen (g/L)	2.62 (2.28-2.98)	2.62 (2.29-3.05)	0.435†
TG (mmol/L)	1.42 (1.04-1.84)	1.42 (0.99-2.16)	0.130†
Lymphocyte count (10^9/L)	1.71 (1.23-2.05)	1.74 (1.36-2.15)	<0.001*†
HDL-C (mmol/L)	1.09 (0.88-1.36)	1.17 (0.96-1.43)	<0.001*†
LDL-C (mmol/L)	2.76 (2.22-3.05)	2.81 (2.26-3.35)	<0.001*†
Uric acid (μmol/L)	307.9 (248.9-370.0)	322.4 (263.6-394.2)	<0.001*†

Neutrophil count (10^9/L)	4.22 (3.52-6.43)	3.79 (2.97-4.95)	<0.001*†
C-reactive protein (mg/L)	6.49 (3.24-15.25)	7.34 (3.81-14.19)	0.162†
Apolipoprotein A-I (g/L)	1.12 (0.96-1.28)	1.18 (1.00-1.43)	<0.001*†
Apolipoprotein B (g/L)	0.87 (0.72-1.00)	0.89 (0.73-1.08)	0.001*†
TC (mmol/L)	4.61 (3.99-5.04)	4.82 (4.11-5.51)	<0.001*†
HCY (µmol/L)	11.85 (10.05-14.13)	11.91 (9.96-14.25)	0.984†
eGFR (mL/min/1.73m^2)	95.4 (78.1-112.4)	92.7 (77.4-110.4)	0.080†
Creatinine (µmol/L)	65.69 (53.95-78.39)	67.30 (55.94-79.48)	0.005*†
Drugs			
Statin, n(%)	515 (50.9)	524 (25.9)	<0.001*‡

Data are represented as the median (interquartile range) for variables and as n (%) for categorical variables.
*P <0.05
†Mann–Whitney U test
‡Chi-square test

Table 2. Binary logistic regression analysis for MMD and Lp(a) concentration

Group	Unadjusted model		Model 1†		Model 2‡	
	OR (95% CI)	P value	OR (95% CI)	P value	OR (95% CI)	P value
Q1 (n=759)	Reference		Reference		Reference	
Q2 (n=759)	1.247 (1.000-1.554)	0.050*	1.251 (1.003-1.560)	0.047*	1.244 (0.995-1.554)	0.055
Q3 (n=760)	1.458 (1.173-1.813)	0.001*	1.443 (1.159-1.795)	0.001*	1.449 (1.161-1.808)	0.001*
Q4 (n=758)	1.613 (1.299-2.002)	<0.001*	1.598 (1.286-1.986)	<0.001*	1.661 (1.330-2.074)	<0.001*
P for trend		< 0.001*		<0.001*		<0.001*

*P <0.05

†Adjustment of Model: BMI and SBP

‡ Adjustment of Model 2: Model 1 plus CRP, HCY, LDL-C, and TG

Manuscript(11).pdf

ORIGINALITY REPORT

9%
SIMILARITY INDEX

PRIMARY SOURCES

1	lipidworld.biomedcentral.com<br>Internet	41 words — 1%
2	www.mdpi.com<br>Internet	39 words — 1%
3	"Minutes of The 43rd General Assembly of The European Association for The Study of Diabetes", Diabetologia, 2008<br>Crossref	34 words — 1%
4	www.frontiersin.org<br>Internet	32 words — 1%
5	www.dovepress.com<br>Internet	25 words — 1%
6	Lingjun Rong, Na Luo, Yanping Gong, Hui Tian, Banruo Sun, Chunlin Li. "One-hour plasma glucose concentration can identify elderly Chinese male subjects at high risk for future type 2 diabetes mellitus: A 20-year retrospective and prospective study", Diabetes Research and Clinical Practice, 2021<br>Crossref	19 words — < 1%
7	repository.ubn.ru.nl<br>Internet	14 words — < 1%

8	"The 34th Great Wall International Congress of Cardiology Asian Heart Society Congress 2023", Cardiovascular Innovations and Applications, 2023<br>Crossref	13 words — < 1%
9	B. Ovbiagele, J. L. Saver, H. Bang, L. E. Chambless, A. Nassief, J. Minuk, J. F. Toole, J. R. Crouse. "Statin treatment and adherence to national cholesterol guidelines after ischemic stroke", Neurology, 2006<br>Crossref	12 words — < 1%
10	nts.prolekare.cz<br>Internet	12 words — < 1%
11	Galina Zemtsovskaja, Jelena Abina, Kalju Meigas, Kristjan Pilt, Margus Viigimaa. "Pulse wave velocity and its gender-related associations with cardiovascular risk factors in a high cardiovascular risk population", Archives of Medical Science - Atherosclerotic Diseases, 2018<br>Crossref	11 words — < 1%
12	arthritis-research.biomedcentral.com<br>Internet	11 words — < 1%
13	d.docksci.com<br>Internet	11 words — < 1%
14	spiral.imperial.ac.uk<br>Internet	11 words — < 1%
15	www.researchgate.net<br>Internet	11 words — < 1%
16	En-zhi JIA. "Serum sodium concentration is significantly associated with the angiographic characteristics of coronary atherosclerosis", Acta Pharmacologica Sinica, 8/2007	10 words — < 1%

Crossref

17 Kang H. Zheng, Benoit J. Arsenault, Yannick Kaiser, Kay-Tee Khaw et al. "apoB/apoA-I Ratio and Lp(a) Associations With Aortic Valve Stenosis Incidence: Insights From the EPIC-Norfolk Prospective Population Study", Journal of the American Heart Association, 2019 — 10 words — < 1%
Crossref

18 academic.oup.com — 10 words — < 1%
Internet

19 research-management.mq.edu.au — 9 words — < 1%
Internet

20 www.cmaj.ca — 9 words — < 1%
Internet

21 Cai-Yan Cui, Tao Ye, Lian-Chao Cheng, Lin Tong, Lan Tong, Zhen Zhang, Lin Cai. "Lipoprotein a Combined with Fibrinogen as an Independent Predictor of Long-Term Prognosis in Patients with Acute Coronary Syndrome: A Multi-Center Retrospective Study", Journal of Cardiovascular Development and Disease, 2022 — 8 words — < 1%
Crossref

22 clin-lab-publications.com — 8 words — < 1%
Internet

23 obgyn.pericles-prod.literatumonline.com — 8 words — < 1%
Internet

24 pure.rug.nl — 8 words — < 1%
Internet

25 worldwidescience.org — 8 words — < 1%
Internet

26 www.pagepressjournals.org
Internet

8 words — < 1%

27 Kanoke, Atsushi, Miki Fujimura, Kuniyasu Niizuma, Akira Ito, Hiroyuki Sakata, Mika Sato-Maeda, Yuiko Morita-Fujimura, Shigeo Kure, and Teiji Tominaga. "Temporal profile of the vascular anatomy evaluated by 9.4-tesla magnetic resonance angiography and histological analysis in mice with the R4859K mutation of RNF213, the susceptibility gene for moyamoya disease", Brain Research, 2015.
Crossref

7 words — < 1%

28 Wei Hu, Wan Jiang, Li Ye, Yanghua Tian, Bing Shen, Kai Wang. "Prospective evaluation of the diagnostic value of plasma apelin 12 levels for differentiating patients with moyamoya and intracranial atherosclerotic diseases", Scientific Reports, 2017
Crossref

7 words — < 1%

29 synapse.koreamed.org
Internet

4 words — < 1%

EXCLUDE QUOTES	OFF	EXCLUDE SOURCES	OFF
EXCLUDE BIBLIOGRAPHY	ON	EXCLUDE MATCHES	OFF
