# Supplementary material for: High lipoprotein(a) concentration is associated with moyamoya disease
Source: Lipids Health Dis. 2024 Jan 22;23:21. doi: 10.1186/s12944-024-02015-1 (PMC10802057; doi:10.1186/s12944-024-02015-1)
Supplement: Supplementary file 2 — Supplementary Material 2 [file 12944_2024_2015_MOESM2_ESM.docx]

**High lipoprotein(a) concentration is associated with moyamoya disease**

Xinyue Chen, MB^1,2#^, Chenxin Song, MB^1,2#^, Xianrun Ma, MB^3^, Junjie Tao, MB^2^, Lijuan Hu, MD^4^, Yuan Xu, MB^5^, Yingping Yi, MD^5^, Xinlei Yang, MD^6*^, Long Jiang, MD, PhD^1*^

^#^ The authors equally contributed to this work

*Co-corresponding authors

**Supplementary Data**

**Table S1 Correlation between lipoprotein(a) and the variables**

**Table S2 Binary logistic regression analysis for MMD and Lp(a) in sensitivity analysis which adjust for statin**

**Table S3 Binary logistic regression analysis for MMD and Lp(a) in sensitivity analysis which adjust for ASCVD**

**Table S4 Binary logistic regression analysis for MMD and Lp(a) in sensitivity analysis which adjust for time of inclusion**

**Table S5 The stratified analysis after adjusting for LDL-C for Lp(a)-cholesterol content, assuming either 17.3%, 30% or 45% mass**

**Fig. S1 Serum lipoprotein(a) level distribution in moyamoya disease group**

**Fig. S2 Serum lipoprotein(a) level distribution in control group**

**Fig. S3 A linear relationship between lipoprotein(a) and moyamoya disease risk by a restricted cubic spine (RCS)**

**Table S1 Correlation between lipoprotein(a) and the variables**

|  | r-cofe | *P*-value |
| --- | --- | --- |
| †Weight (n=3036) | -0.092 | <0.001* |
| †SBP (n=3036) | -0.019 | 0.291 |
| †LDL-C (n=3036) | 0.142 | <0.001* |
| †HCY (n=3036) | 0.014 | 0.457 |
| †Uric acid (n=3036) | -0.065 | <0.001* |
| †TC (n=3036) | 0.079 | <0.001* |
| †TG (n=3036) | -0.060 | 0.001* |
| †Fibrinogen (n=3036) | 0.154 | <0.001* |
| †Glucose (n=3036) | -0.038 | 0.038* |
| †latelet count (n=3036) | 0.048 | 0.008* |
| †Apolipoprotein A-I (n=3036) | -0.021 | 0.251 |
| † Apolipoprotein B (n=3036) | 0.116 | <0.001* |
| †CRP (n=3036) | 0.007 | 0.712 |
| †Lymphocyte count (n=3036) | -0.066 | <0.001* |
| †Neutrophil count (n=3036) | 0.040 | 0.028* |
| †NLR (n=3036) | 0.073 | <0.001* |

*CRP* C-reactive protein, *HCY* homocysteine, *LDL-C* low density lipoprotein cholesterol, *NLR* neutrophil-lymphocyte ratio, *SBP* systolic blood pressure, *TC* total cholesterol, *TG* triglyceride

* *P* <0.05

†Pearson test

‡Tests were two-sided

**Table S2 Binary logistic regression analysis for MMD and Lp(a) in sensitivity analysis which adjust for statin**

| Group | Unadjusted model |  | Model 3 |  |
| --- | --- | --- | --- | --- |
|  | OR (95% CI) | *P* value | OR (95% CI) | *P* value |
| Q1 (n=759) | Reference |  | Reference |  |
| Q2 (n=759) | 1.247 (1.000-1.554) | 0.050* | 1.260 (1.002-1.584) | 0.048* |
| Q3 (n=760) | 1.458 (1.173-1.813) | 0.001* | 1.412 (1.124-1.775) | 0.003* |
| Q4 (n=758) | 1.613 (1.299-2.002) | <0.001* | 1.524 (1.212-1.916) | <0.001* |
| *P* for trend |  | < 0.001* |  | 0.966 |

*BMI* body mass index, *CI* confidence intervals, *CRP* C-reactive protein, *LDL-C* low density lipoprotein cholesterol, *Lp(a)* lipoprotein (a), *MMD* moyamoya disease, *OR* odd ratios, *SBP* systolic blood pressure, *TG* triglyceride

**P* <0.05

Model 3: adjusted for BMI, SBP, CRP, HCY, LDL-C, TG and statin

**Table S3 Binary logistic regression analysis for MMD and Lp(a) in sensitivity analysis which adjust for ASCVD**

| Group | Unadjusted model |  | Model 1† |  | Model 2‡ |  |
| --- | --- | --- | --- | --- | --- | --- |
|  | OR (95% CI) | *P* value | OR (95% CI) | *P* value | OR (95% CI) | *P* value |
| Q1 (n=708) | Reference |  | Reference |  | Reference |  |
| Q2 (n=708) | 1.186 (0.943-1.491) | 0.145 | 1.194 (0.950-1.503) | 0.129 | 1.194 (0.947-1.506) | 0.134 |
| Q3 (n=708) | 1.436 (1.146-1.800) | 0.002* | 1.429 (1.139-1.792) | 0.002* | 1.445 (1.148-1.819) | 0.002* |
| Q4 (n=708) | 1.602 (1.281-2.005) | <0.001* | 1.596 (1.274-1.998) | <0.001* | 1.671 (1.327-2.104) | <0.001* |
| *P* for trend |  | < 0.001* |  | 0.031* |  | 0.145 |

*ASCVD* arteriosclerotic cardiovascular disease, *BMI* body mass index, *CI* confidence intervals, *CRP* C-reactive protein, *LDL-C* low density lipoprotein cholesterol, *Lp(a)* lipoprotein (a), *MMD* moyamoya disease, *OR* odd ratios, *SBP* systolic blood pressure, *TG* triglyceride

**P* <0.05

†Model 1: adjusted for BMI, SBP

‡Model 2: adjusted for model1 plus CRP, HCY, LDL-C, and TG

**Table S4 Binary logistic regression analysis for MMD and Lp(a) in sensitivity analysis which adjust for time of inclusion**

| Group | Unadjusted model |  |  | Model 1† |  |  | Model 2‡ |  |
| --- | --- | --- | --- | --- | --- | --- | --- | --- |
|  | OR (95% CI) | *P* value |  | OR (95% CI) | *P* value |  | OR (95% CI) | *P* value |
| Q1 (n=510) | Reference |  |  | Reference |  |  | Reference |  |
| Q2 (n=510) | 1.009 (0.772-1.319) | 0.946 |  | 1.016 (0.777-1.328) | 0.909 |  | 1.026 (0.781-1.346) | 0.855 |
| Q3 (n=511) | 1.258 (0.968-1.636) | 0.087 |  | 1.259 (0.968-1.638) | 0.086 |  | 1.308 (1.000-1.711) | 0.050* |
| Q4 (n=509) | 1.437 (1.107-1.865) | 0.006* |  | 1.434 (1.105-1.862) | 0.007* |  | 1.513 (1.156-1.980) | 0.003* |
| *P* for trend |  | < 0.001* |  |  | 0.018* |  |  | 0.025* |

*BMI* body mass index, *CI* confidence intervals, *CRP* C-reactive protein, *LDL-C* low density lipoprotein cholesterol, *Lp(a)* lipoprotein (a), *MMD* moyamoya disease, *OR* odd ratios, *SBP* systolic blood pressure, *TG* triglyceride

**P* <0.05

†Model 1: adjusted for BMI, SBP

‡Model 2: adjusted for model1 plus CRP, HCY, LDL-C, and TG

**Table S5 The stratified analysis after adjusting for LDL-C for Lp(a)-cholesterol content, assuming either 17.3%, 30% or 45% mass**

| Group | Lp(a)-adjusted LDL-C using 17.3% adjustment | | Lp(a)-adjusted LDL-C using 30% adjustment | | | Lp(a)-adjusted LDL-C using 45% adjustment | | |
| --- | --- | --- | --- | --- | --- | --- | --- | --- |
|  | ≤ 130 mg/dL  (n=2502) | >130 mg/dL  (n=534) | ≤ 130 mg/dL  (n=2552) | >130 mg/dL  (n=484) |  | | ≤ 130 mg/dL  (n=2615) | >130 mg/dL  (n=421) |
| Q1 (n=759) | Reference | Reference | Reference | Reference |  | | Reference | Reference |
| Q2 (n=759) | 1.246 (0.980-1.585),  *P*=0.072 | 1.032 (0.566-1.882),  *P*=1.032 | 1.256 (0.989-1.596), *P*=0.062 | 0.926 (0.500-1.717), *P*=0.808 |  | | 1.254 (0.988-1.592), *P*=0.063 | 0.877 (0.463-1.659), *P*=0.686 |
| Q3 (n=760) | 1.428 (1.124-1.814),  *P*=0.004* | 1.304 (0.733-2.320), *P*=0.366 | 1.422(1.120-1.804), *P*=0.004* | 1.280 (0.708-2.314), *P*=0.414 |  | | 1.414 (1.116-1.792), *P*=0.004* | 1.253 (0.679-2.315), *P*=0.471 |
| Q4 (n=758) | 1.588 (1.253, 2.013), *P*<0.001* | 1.248 (0.694-2.245), *P*=1.248 | 1.585 (1.253-2.004), *P*<0.001* | 1.045 (0.551-1.979), *P*=0.893 |  | | 1.602 (1.271-2.021), *P*<0.001* | 0.570 (0.254-1.280), *P*=0.173 |

*BMI* body mass index, *CRP* C- reactive protein, *HCY* homocysteine, *Lp(a)* lipoprotein (a), *LDL-C* low density lipoprotein cholesterol, *SBP* systolic blood pressure, *TG* triglyceride

All subgroups were adjusted for BMI, SBP, CRP, TG, HCY

Data is represented as odd ratios (95% confidence intervals), *P* value

*The stratified test used binary logistic regression with *P* value < 0.05 being significant


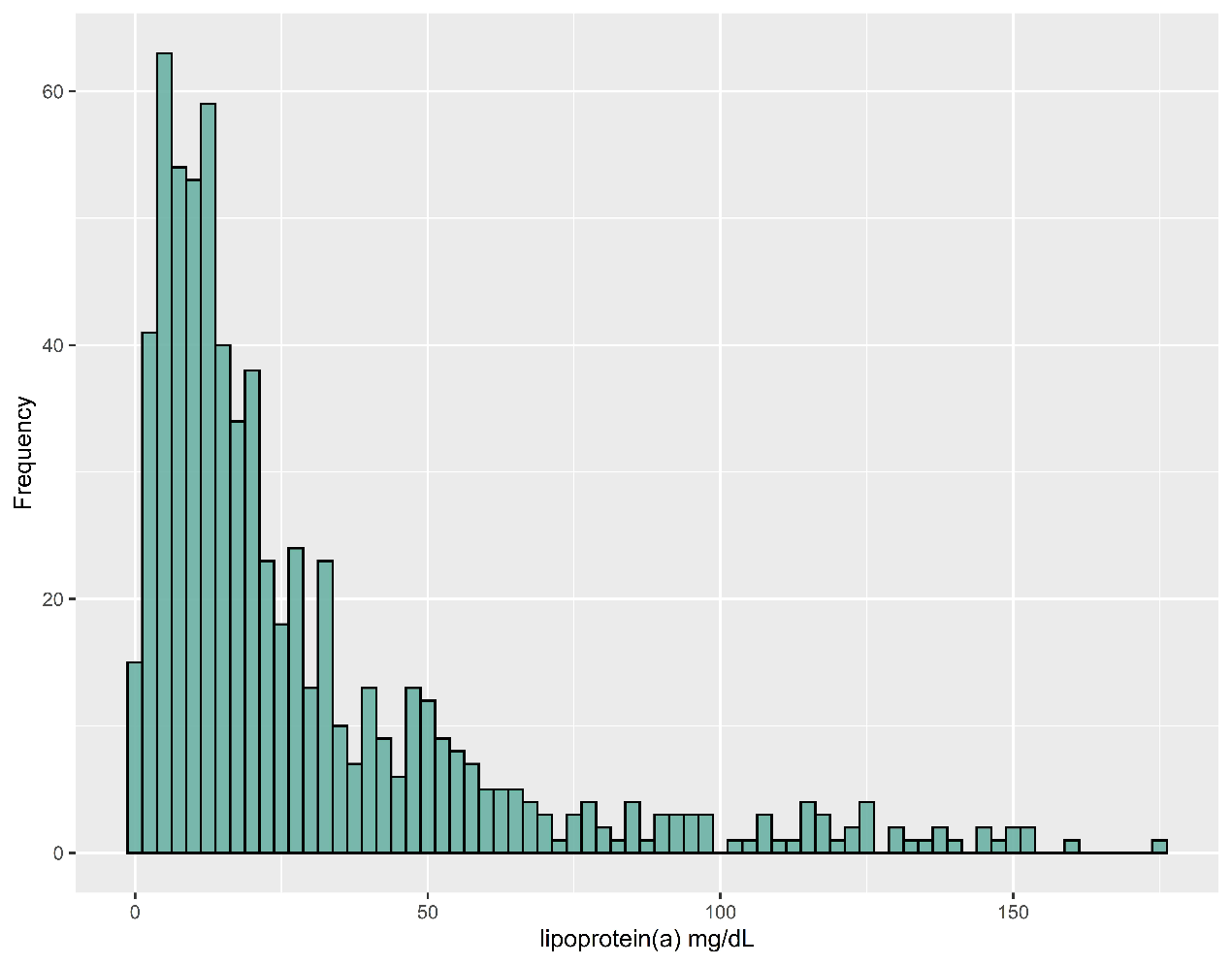


**Fig. S1 Serum lipoprotein(a) level distribution in moyamoya disease group**

**
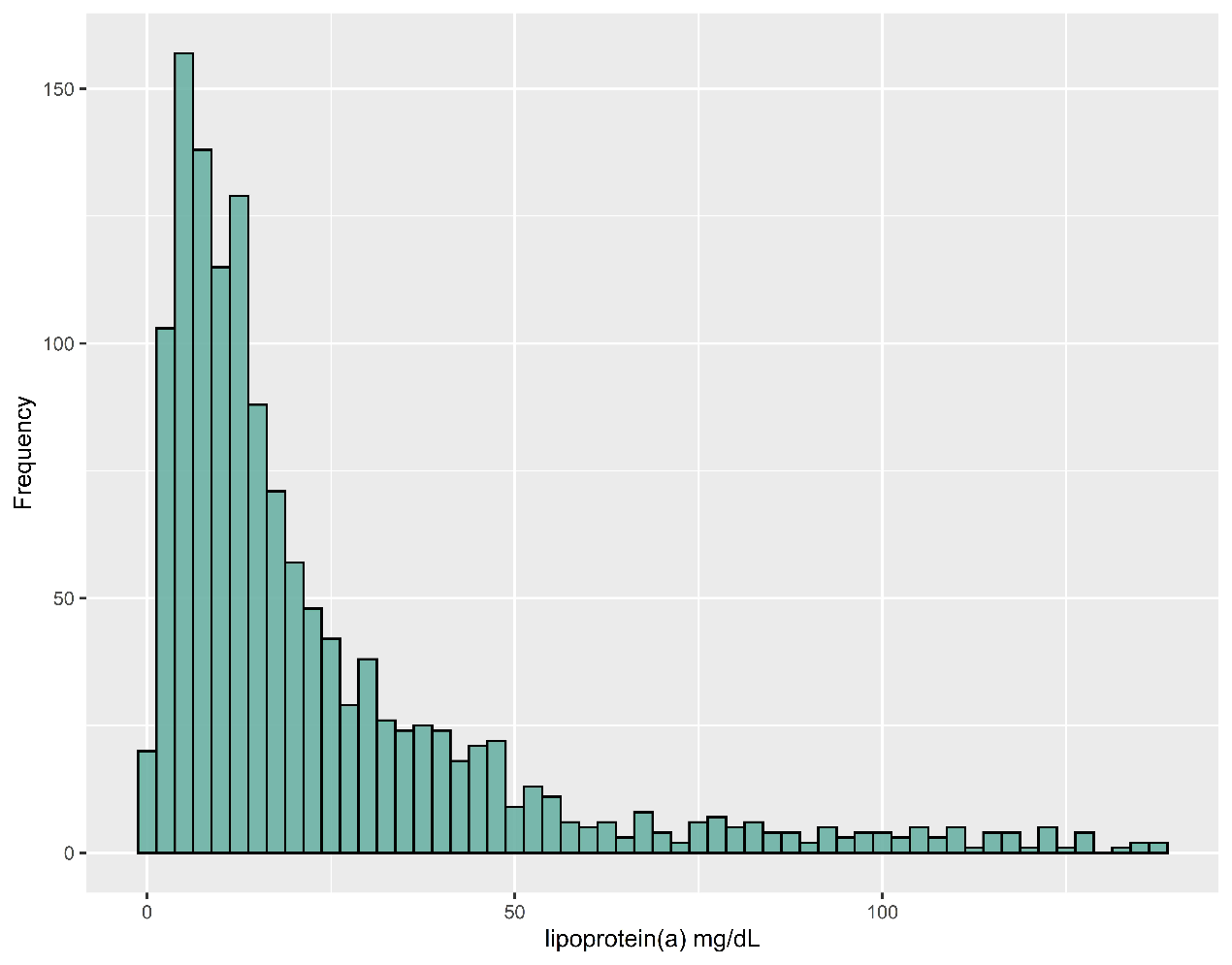
**

**Fig. S2 Serum lipoprotein(a) level distribution in control group**

**
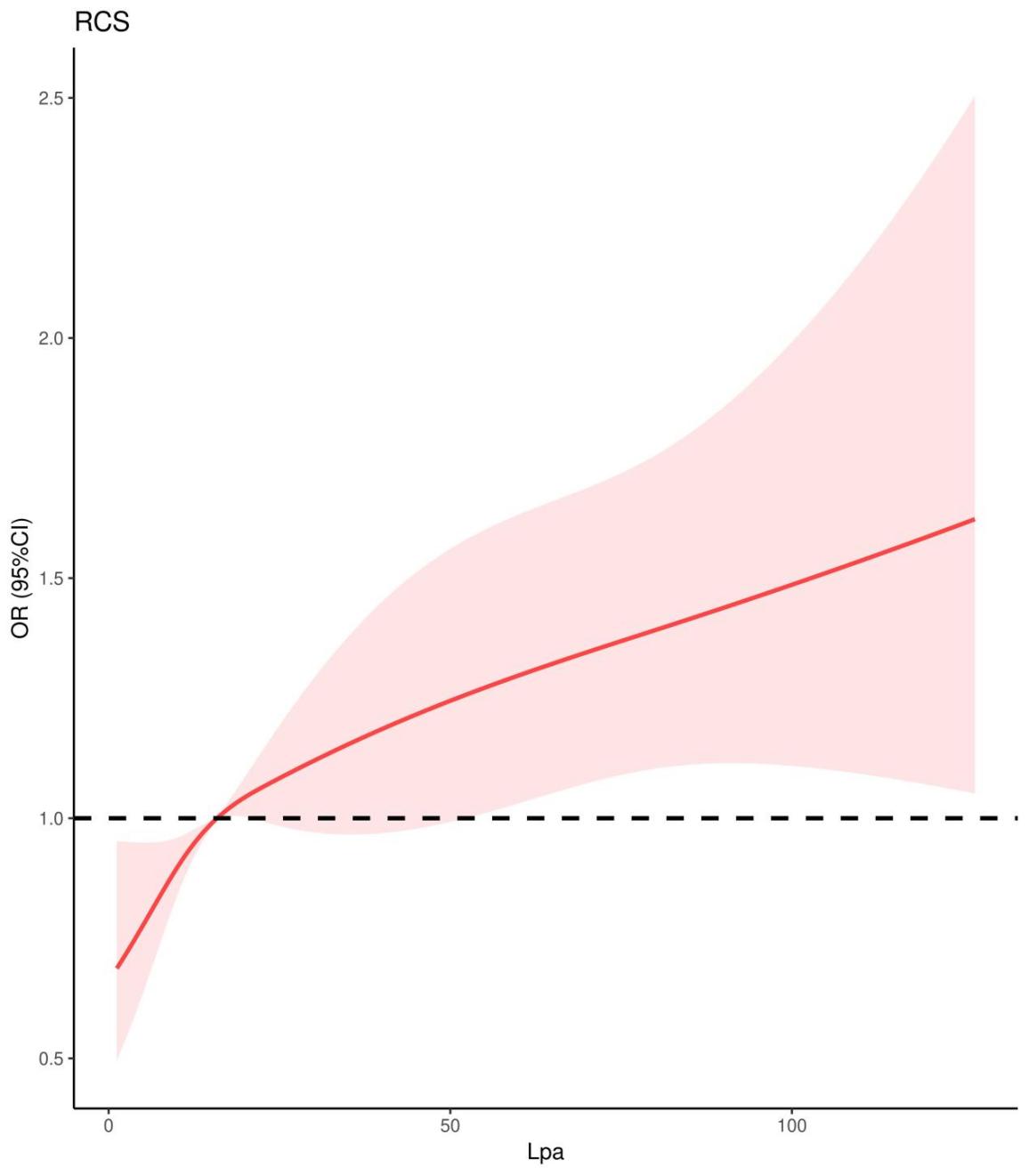
**

**Fig. S3 A linear relationship between lipoprotein(a) and moyamoya disease risk by a restricted cubic spine (RCS)**
